# A Ten Minutes Sermon on Nutrition

**Published:** 1887-04

**Authors:** C. M. Wright

**Affiliations:** Cincinnati


### A Ten Minutes Sermon on Nutrition.

BY C. M. WRIGHT, D.D.S., CINCINNATI.

[Read at the Forty-Third Annual Meeting of the Mississippi Valley Dental Association.]

Don Quixote.—" 'But reach hither thy hand, Sancho, and feel how many teeth are wanting on the right side of my upper jaw; for there I feel the pain.'

Sancho put his finger into Don Quixote's mouth, and feeling about said: 'How many teeth had your worship on this side?'

'Four,' answered Don Quixote, 'beside the eye tooth, all perfect and sound.'

'Think well what you say, sir,' answered Sancho.

'I say four, if not five,' answered Don Quixote; for in my whole life I never had a tooth drawn, nor have I lost one by rheum nor decay.'

'Well then, said Sancho, 'on this lower side your worship has but two and a half; and in the upper, neither half nor whole; all is as smooth and even as the palm of my hand.'

'*Unfortunate that I am!*' said Don Quixote, hearing these sad tidings from his squire; 'I had rather they had torn off an arm, provided it were not the sword-arm ; for thou must know, Sancho, that a mouth without teeth is like a mill without a stone; and that a diamond is not so precious as a tooth.'"

This scene and conversation was recorded by Cervantes just three hundred years ago ; and we learn that the chivalrous Knight of the Sorrowful Figure seemed to appreciate fully, one at least, of the several functions of the teeth, "for a mouth without teeth is like a mill without a stone."

If we could have before our minds a clear and full impression of the alimentary canal of a "higher animal," and could see it work, could follow the entire process from the mouth onwards, could observe all the changes, and all the means of effecting the changes and in the cooked and uncooked products of the animal and vegetable kingdoms which we call food-stuffs, if we could follow these processes as we can the operations of a paper mill, let us say, at one of our expositions, we should perhaps be tempted to cry out with Don Quixote, "that a diamond is not more precious than a tooth," because the teeth are the mill-stones in the continually progressing process of preparing and digesting food-stuffs for such animals.

We spend our lives in repairing and caring for this part, the vestibule, as it were, of the alimentary canal of man, and ought to be able to say, if we conld lay aside all interested considerations, just how precious a tooth is; and I think we can say that perhaps no part of the man's apparatus for the reduction and solution, or *digestion* of food-stuffs could be so easily dispensed with or replaced by other means so easily as the teeth. A tooth, then, is not as valuable as a diamond, unless it is beautiful in shape and position, and assists in producing agreeable expressions of the passing emotions of the mind, as recorded in the human face; and unless at the same time that it is a thing of beauty, is also a stone in the mill—useful in the economy. The modern dentist has been accused lately by a writer for a medical journal of having become so imbued with the idea of the importance of "saving every tooth," or rather of not extracting any tooth that can be made *tolerable* in its socket, that he fails to observe the broader view of the health of the entire body, in his narrow view of his own art. He fails to observe that a tooth may be a means of injurious irritation, producing reflex troubles in the organism, these troubles manifesting themselves frequently in the special sense organs of the neighborhood—the eye and the ear.

The accusation is in the main true against the better class of dentists, and possibly similar accusations might be brought against all so-called "specialists." There certainly is a tendency of the human mind to magnify the importance of things which occupy its special attention. We do, no doubt, too often forget that the natural teeth of man can be dispensed with without perceptible detriment to the general health, and that often it were better that one tooth should be cast out rather than that the function of the entire organ should be destroyed To stick to Don Quixote's simile about the mill-stone, we might say that a mill-stone with a flaw is of no value, and that the miller should rather try to pulverize his corn in a mortar with a pestle, or with a hammer, than by the use of the imperfect mill-stone.

We are prone to forget, in the study of our fine methods, what we should wish to accomplish. We must not forget that a handsome artificial crown, set in the most ingenious of our many methods upon a tooth root, is of no value, it that root has severed its friendly relations with the central nervous system of the body, so that it cannot call for an extra blood supply in case of need, as even healthy tissue can ; if it cannot set the vaso-motor nerves at work, and influence the entire circulation and the action of the heart, and prove that it is still a part of the organism, and even if it could do this, and when pabulum is supplied by other than perfectly natural stimuli, if it is so weak, so degenerate that on such an occasion it could not take up the food offered to it and satisfy itself with it, and give off its proper waste, but must remain like a thorn in the flesh, of what beauty or value is the new method of our art?

The tooth is a living part of a living body. It is a part of the organism, and it is under the same laws that govern all living matter, whether that living matter be found in a seed of a plant, an amœba, a bacteria, or the brain of a man. Each atom of living matter in the universe, whether it be found floating in an apparent independence in the air about us, or is dredged from the sea, is subject to the laws of all living matter—is subject to the laws of nutrition. There are three things indispensable to what we call nutrition, whether of the living atom, or the complex organism of the most perfect animal. These three things are:

1st. A supply of proper pabulum.

2d. The condition of the organism to be able to satisfy itself, to appropriate, to take in this pabulum or the power of assimilation.

3d. The means or power of getting rid of the results of this metabolism—the waste, the ashes, the effects of its own activity.

We know how true this is in regard to an animal. We know that the study of anatomy and physiology seems to be only a study of apparati for the taking in and dissolving and circulating and burning up vegetable and animal products, and the getting rid of the waste, the results of the combustion, and that the sole object of the organism seems to be, to seek for, and take in from earth and air, food; to burn up this food in its tissues, and to return the products to the earth and air. It seems to be the sole design of all the beautiful arrangements of the organs and tissues even of man to enable him simply to eat, drink, be merry, and die. When we follow the life history of a little jelly-like speck of living matter, too minute to be observed excepting by powerful magnifying lenses, we observe the same thing. The little jelly-like speck, eats, drinks, is merry, and dies, leaving other little jelly-specks to follow the same everlasting destiny.

We have come to regard the science of medicine as based upon these facts. Its physiology is the proper action of organisms in reference to normal income and normal outgo. (In a physiological state every tissue, as well as the entire body is merry.) Its pathology is simply a variation, more or less, in reference to the proper action of these things, and its thereapeutics, is an attempt to regulate the income and outgo. While this is the law for the whole body, it is the law for every part, and the specialist who proposes to attend to the pathology and therapeutics of a part, can no more cut himself off and say, "I heal the ear, the eye, the tooth, and let the general practitioner care for the body," than could the rudder of ship say " I turn whichever way I please, absolutely independent of the general motion of the ship. Let the engine work the propeller as it pleases; let the wind blow against the sails as it listeth; they, too, are specialists (brought up in different schools). I, as the rudder, will take care of my own department." We can no more separate the tooth from the laws of nutrition and retain it in the tissues of the body, than we can expect a glass tooth or a leaden tooth to be brought under these same laws, and attain the power of assimilation, no matter where it might be implanted in the tissues.

The tooth then being a living body, capable of taking up pabulum, using this pabulum for its own benefit, and giving off its waste, like every other tissue, must remain in physiological relations with the entire organism, and it is our business to maintain these relations between the tooth and the entire body, if possible. When harmony is lost beyond our power to recover it, when from mal-position of one tooth, or an entire set, or from any cause, the beauty of the patient is destroyed, or the use of the set of teeth as an organ of mastication is destroyed, a tooth or a set of teeth is of no more value than a rudder which cannot be made to answer the steersman's wheel. Cut it off then, and depend upon the compensating energy of the engine and the sails! This is our work, and it is a great one, if we do not take too narrow a view of it.

We grope about in the twilight. We do not see clearly what goes on just below the surface. We are not settled in our opinions about the morphology of the tissues, but we have seen some things which teach us that when we cut into the dentine of a tooth with our engines or excavators, even though the sense of pain has not been awakened, we have made an impression upon the central nervous system which will call into counsel the heart and every tissue of the body, and measures will be taken, assisted by every tissue of the body, to protect that tooth from the threatened danger.

The apparently slight irritation of a peripheral pin-head excavation in the process of dental caries on a molar tooth, though giving no notice in the sensorium of the man, will call out increased nutritive energies of the dental pulp, and it immediately begins to protect itself, as it were, with the products of the increased activity. Who would call this a purely local affair? Has the general circulation not been affected? Have the nervous centers no hand in the affair? Have the remotest tissues of the body not sympathized with the tooth? What whispers has the blood carried in its flying visit to the living cells of other tissues?

A delicate instrument, capable of marking every change in income and outgo, would show a variation in the nutrition of the whole body on account of the little "dental caries" affecting one molar tooth in this strong man's mouth, for where does the tooth get its nourishment if not from the food which it helps to grind, and which passes on to the embraces of the solvents in the different parts of the factory.

Two or three years ago a dispute arose in this very society in regard to the question of whether "dental caries" could be called a *disease* of the teeth or not. May I be pardoned for making the apparently dogmatic assertions, that "dental caries" in a living tooth is a disease which, without reference to its etiology, affects not only the tooth but more or less the entire organism, and that the dentist of the future will, when examining a simple "*cavity*" in a tooth, have before his mind's eye the entire panorama of the dependence of one tissue of a complex organism upon every other tissue, in its relation to nutrition. The dentist of the future will know that when he operates upon a tooth for the removal of injurious deposits about the neck, that his object is to place the living tooth in a condition of harmony with the nutritive functions of the entire organism, and that the last simple mechanical operation of "scaling the teeth is just so much of a local remedy as is the administration of aconite as a cardiac depressant, and no more.

We may have separate schools for special instruction in our art. We may have separate journals for the dissemination of views relating to our art, the subject matter of which cannot be of any more interest to the general practitioner of medicine than would detailed accounts of obstetrical cases be to us; yet, the science and art of dentistry can have no other foundation upon which to rest than that which must support "general medicine." The gynecologist falls into routine ways of practice as does the dentist, but biology, in its departments of morphology and physiology, *must* form the foundation of both the *arts*.

